# 

**Published:** 1884-11

**Authors:** 


CONTENTS.

| | PAGE. |
|---|---|
| Digestion | 199 |
| Object of Eating | 201 |
| Milk Diet | 202 |
| Substances in the Nose | 203 |
| Sanitary Science | 204 |
| Long Life | 205 |
| Extreme Minuteness | 207 |
| Coated Tongues | 207 |
| Renovating Old Oil Paintings | 209 |
| Historical Hot Summers | 209 |
| The Drowning Season | 210 |
| Stone or Gravel | 211 |
| How We Squander Health | 212 |
| The Whip and Spur | 213 |
| The Teeth | 213 |
| Diphtheria | 214 |
| Children | 217 |

